# Two new species of Lobellini from Tianmu Mountain, China (Collembola, Neanuridae)

**DOI:** 10.3897/zookeys.726.11934

**Published:** 2018-01-02

**Authors:** Ji-Gang Jiang, Wen-Bin Wang, Hu Xia

**Affiliations:** 1 College of Life and Environmental Sciences, Hunan University of Arts and Science; Collaborative Innovation Center for Efficient and Health Production of Fisheries in Hunan Province, Changde 415000, Hunan, China; 2 Department of Life Science and Technology, Central South University of Forestry and Technology, Changsha 410004, Hunan, China

**Keywords:** *Crossodonthina bidentata*, *Lobellina fusa* sp. n., *Paralobella tianmuna* sp. n., Neanurinae, taxonomy

## Abstract

Three species of the subfamily Neanurinae (Collembola: Neanuridae) are recorded from Tianmu Mountain, Zhejiang Province, east China. Two of them, *Lobellina fusa* **sp. n.** and *Paralobella tianmuna* **sp. n.**, are new to science and described in this paper. *Lobellina fusa* **sp. n.** can be recognized by the presence of six teeth on mandible and the fusion of dorsointernal tubercles on the head. *Paralobella tianmuna* **sp. n.** is characterized by a mandible with seven teeth, the lateral tubercle of Abd. II–III respectively with 7 (6+s) chaetae. *Crossodonthina bidentata* Luo & Chen, 2009 is widely distributed in the mountain from 300 to 1500 m a.s.l.

## Introduction

To date, on a worldwide scale, the tribe Lobellini consists of 17 genera (including two subgenera) and approximately 157 species mainly from South-East Asia and the Australian-Oceania region (Bellinger et al. 2017). Up to now, only six genera and 12 species were reported from mainland China (Denis 1929; Stach 1964; Yue and Yin 1999; Wang 2003; Xiong et al. 2005; Ma and Chen 2008; Luo and Chen 2009; Jiang and Zhang 2012; Jiang et al. 2012; Luo and Palacios-Vargas 2016; Wang et al. 2016). The tribe is diversified in all regions sampled so far, but huge areas have never been sampled and the knowledge of Chinese fauna of Lobellini can be considered as very incomplete.

Tianmu Mountain, located in Lingan City, Zhejiang Province, east China, covers an area of 4300 hectares. The elevation of the highest peak of the mountain is more than 1500 meters. It belongs to the subtropical humid monsoon climate zone. The flora is a typical subtropical evergreen broad-leaved forest. One of the main targets of the Zhejiang Tianmu Mountain National Nature Reserve is the protection of rare and endangered plants, such as *Ginkgo biloba*, *Cercidiphyllum japonicum*, and *Liriodendron chinensis*. Till now, more than 4000 species (including 657 type species) of insects were reported from the mountain (Wu and Pan 2001). However, the Collembolan fauna of the mountain is poorly known, and only very few neanurid species were reported from it (Luo and Chen 2009, Jiang et al. 2012). In 2011, organized by The Management Bureau of Zhejiang Tianmu Mountain National Nature Reserve, we carried out field work in this mountain. Three species of the tribe Lobellini were identified and two of them are described as new.

## Terminology

The terminology and layout of the tables used in this paper follow Deharveng (1983), Deharveng and Weiner (1984), and Smolis and Deharveng (2006).

### Abbreviations used

**General morphology**:

**Abd** abdomen,

**Ant** antenna,

**Cx** coxa,

**Fe** femur,

**Scx2** subcoxa 2,

**Ti** tibiotarsus,

**Th** thorax,

**Tr** trochanter,

**VT** ventral tube.

**Groups of Chaetae**:

**Ag** antegenital,

**An** anal,

**Fu** furcal,

**Ve** ventroexternal,

**Vi** ventrointernal,

**Vl** ventrolateral,

**De** dorsoexternal,

**Di** dorsointernal,

**Dl** dorsolateral,

**L** lateral,

**Oc** ocular,

**So** subocular.

**Tubercles**:

**An** antennal,

**Fr** frontal,

**Cl** clypeal,

**Types of chaetae**:

**Ml** long macrochaeta,

**Mc** short macrochaeta,

**Mcc** very short macrochaeta,

**me** mesochaeta,

**mi** microchaeta,

**i** microchaeta,

**ms** s-microchaeta,

**s** s-chaeta,

**or** organite of Ant. IV,

**i** small ordinary chaeta on Ant. IV,

**mou** thin cylindrical chaetae on Ant. IV (“soies mousses”),

**x** labial papilla x.

## Materials and methods

All specimens were collected with the aid of Tullgren funnels or aspirators, and preserved in 95% ethanol. They were cleared in Nesbitt’s fluid and mounted on slides in Hoyer’s medium. Preparations were dried for 7–10 days in oven at 55 °C, and then ringed with lacquer. The morphological characters were observed and figures were drawn using a phase contrast microscope Nikon 80i. Material is deposited in the Key Laboratory of Zoology, Hunan University of Arts and Science, Changde, Hunan Province, China.

## Taxonomy

### Tribe Lobellini Cassagnau, 1983

#### Genus *Crossodonthina* Yosii, 1954

##### Crossodonthina bidentata

Taxon classificationAnimaliaCollembolaNeanuridae

Luo & Chen, 2009

###### Material.

Three males and two females, on the path from Qili Pavilion to Longfengjian, Tianmu Mountain, Zhejiang Province, China. Coordinates: 30°20'36"N, 119°26'17"E, 800–1050 m a.s.l., 26 July 2011, leg. Ji-Gang Jiang (collection number: J2011072601). Ten males and eight females, Zhonglie Temple, Tianmu Mountain, Zhejiang Province. Coordinates: 30°23'03"N, 119°26'20"E, 300–400 m a.s.l., 24 July 2011, leg. Ji-Gang Jiang (collection number: J2011072401). Four males and two females, in bamboo forest, Hengwu, Tianmu Mountain, Zhejiang Province. Coordinates: 30°19'01"N, 119°26'15"E, 400–500 m a.s.l., 27 July 2011, leg. Ji-Gang Jiang (collection number: J2011072703.

###### Short redescription.

Eyes 2+2. Labrum truncate and chaetal formula as 2/5, 2. Mandible consisting of two basal teeth and three rami. Chaeta O of cephalic tubercle Fr present. Body macrochaeta acuminate and ciliate. Formula of dorsal sensilla on thorax and abdomen as 0, 2+ms, 2/1, 1, 1, 1, 1, 0. Unguis with one inner tooth and no lateral teeth. Unguiculus absent. Di tubercles on Abd. V fused on the axis.

###### Remarks.

*Crossodonthina bidentata* is widely distributed in the mountain from 300 to 1500 m, living in decayed leaves of woody plants as well as bamboo.

#### Genus *Lobellina* Yosii, 1956

##### Lobellina fusa sp. n.

Taxon classificationAnimaliaCollembolaNeanuridae

http://zoobank.org/0D0D9A9D-5159-48B6-BEC0-AE0976E274FB

**Figures 1–4.**
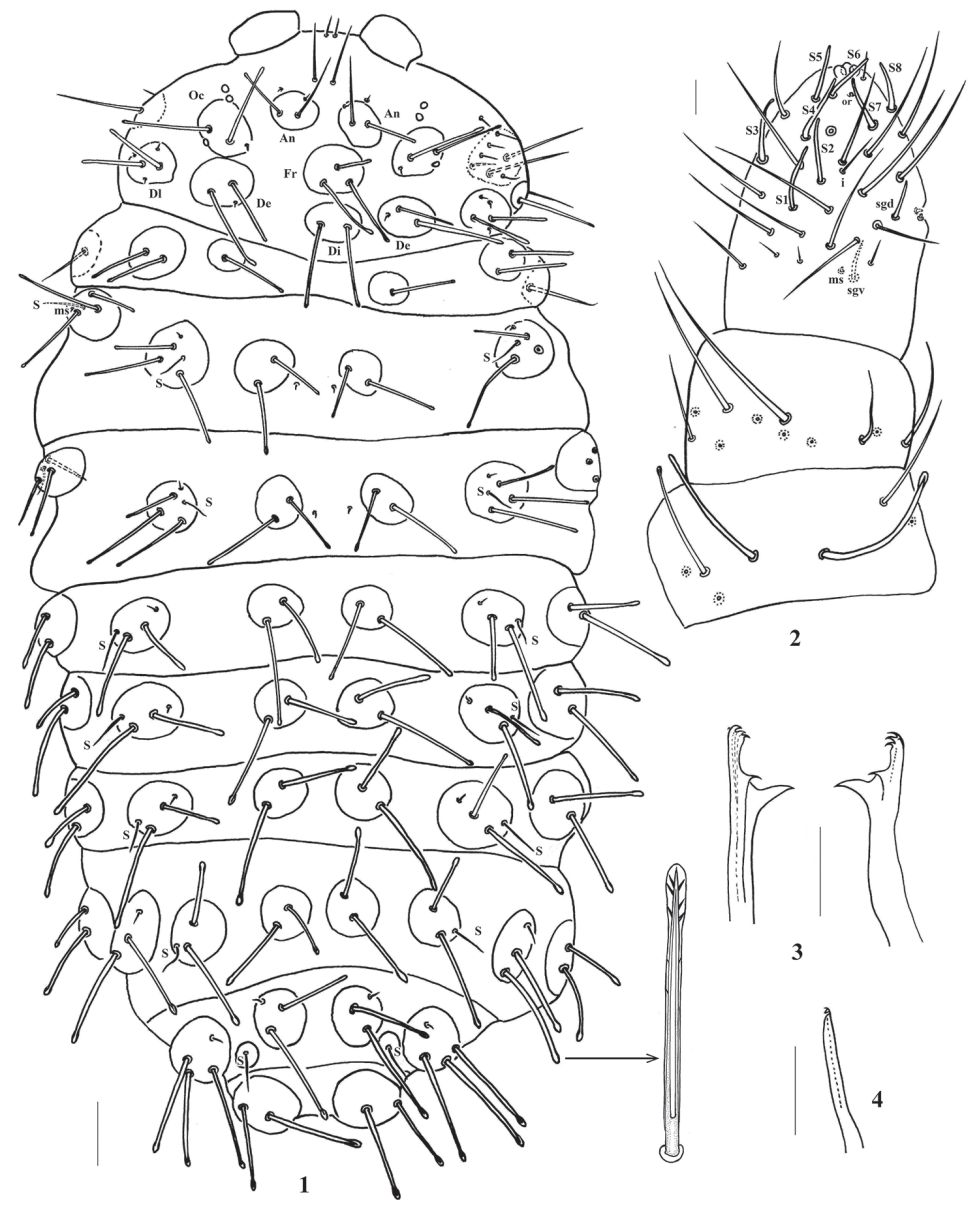
*Lobellina fusa* sp. n. **1** Dorsum of body **2** Antenna **3** Mandible **4** Maxilla. Scale bars: 100 μm (**1**); 20 μm (**2–4**).

**Figures 5–7.**
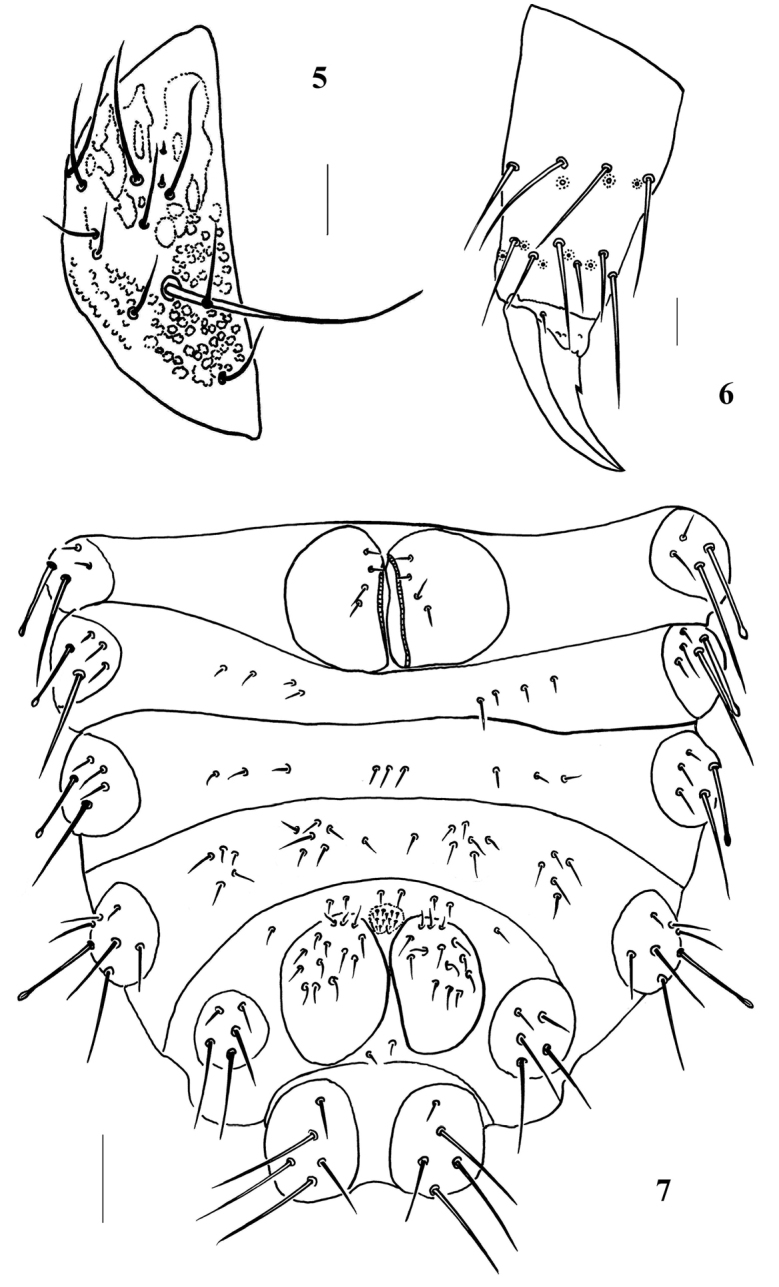
*Lobellina fusa* sp. n. **5** Labium **6** Tibiotarsus and claw of hind leg **7** Ventral side of Abdomen. Scale bars: 20 μm (**5–6**); 100 μm (**7**).

Figs 1–4 , 5–7 , Appendix 1 , Tables 1a –1b

###### Type material.

Holotype male on slide, on the path from Longfengjian to Fairy Peak, Tianmu Mountain, Zhejiang Province, China. Coordinates: 30°23'11"N, 119°26'07"E, 1100–1500 m a.s.l., 25 July 2011, leg. Ji-Gang Jiang (Housed in Hunan University of Arts and Science, J2011072501). Paratypes: three females and two males, same data as holotype (Housed in Hunan University of Arts and Science, J2011072501).

###### Etymology.

The species name refers to the fused Di tubercles on head.

###### Diagnosis.

Habitus typical of the genus *Lobellina*. Body dorsal tubercles well developed. Cephalic Di tubercles fused. Chaeta O of tubercle Fr present. 3+3 black eyes. Labrum chaetotaxy as 0/2, 2. Mandible with six teeth. Maxilla consisting of two crochet-like lamellae and two teeth. Chaetae formula of tubercle Di on Th. I–Abd. V as 1, 3, 3/2, 2, 2, 2, 3. S-chaetae and s-microchaeta formula on Th. I–Abd. V as 0, 2+ms, 2/1, 1, 1, 1, 1. Ventral tube with 4+4 chaetae, furcular vestige with 3 chaetae. Unguis with an inner tooth and without lateral tooth.

###### Description.

Body length 2.2–3.7 mm. Body color red while alive and white in alcohol.

*Head*: eyes 3+3, black (Fig. 1). Antenna 4-segmented. Ant. I and II with seven (two with swollen apex) (Fig. 2) and eleven chaetae respectively. Ant. III dorsally fused to Ant. IV. Ant. III organ consists of two short rods and two long guard chaetae, two short rods exposed in separate pits. Ant. IV with trilobed apical bulb, dorsally with eight sensilla, slender *i* chaeta, 12 slender cylindrical chaetae (mou) and minute organite (or) (Fig. 2). Labrum chaetotaxy as 0/2, 2. Labium with eleven chaetae and two minute distal x sensilla (Fig. 5). Mandible with three main teeth, the apical one subdivided in four minute toothlets (Fig. 3). Maxilla consists of two fused lamellae, stylet-like and one with two small apical teeth (Fig. 4). Cephalic tubercles and chaetotaxy shown on Fig. 1 and Appendix 1, Table 1a. Macrochaetae and mesochaetae on body weakly serrate (observe under lens of 100×), and sheathed on distal half, ending in a swollen and blunt apex (Fig. 1). Microchaetae smooth and pointed. All dorsal cephalic tubercles (except Di) independent.

*Thoracic and abdominal tubercles and chaetotaxy* shown in Figs 1, 7 and in Appendix 1, Table 1b. Sensory chaetae and s-microchaetae formula on Th. I–Abd. V as 0, 2+ms, 2/1, 1, 1, 1, 1. Chaetae Di3 free on Th. II and III. Each tubercle on Abd. VI with 7 chaetae (3 Ml and 4 Mc or me).

*Appendages*: Unguis with an inner tooth and without lateral tooth (Fig. 6). Unguiculus absent. Ventral tube with 4+4 chaetae (Fig. 7), furcular vestige with three chaetae and no microchaetae (Fig. 7). Chaetotaxy of legs, ventral tube, and furcular remnant shown in Appendix 1, Table 1b.

###### Ecology.

Under leaves in forest.

###### Remarks.

The taxon *Lobellina* was erected by Yosii in 1956 as a subgenus of the genus *Lobella* Börner, 1906. It was raised to generic status by Cassagnau (1983) and redefined by Deharveng and Weiner (1984): body without blue pigment, 3+3 black eyes. Tubercles on the head and the tergites well developed, marked by a bump of the tegument and /or by differentiated tertiary grain, or some more strong secondary grains. Body without reticulation. Chaetotaxy of labrum as 0/2, 2. Maxilla styliform, mandible tridentate to multidentate. The sgd of Ant. III organ not shifted to ant. IV. Dorsal macrochaetae thickened and double lined, rounded to the apex. Chaetotaxy of type s normal (2+ms, 2/1, 1, 1, 1, 1). Abd. I without supplemental s-chaeta on lateral tubercle. Posterior chaetotaxy of head of cross-type. Chaetotaxy of tubercles Di of Th. II and III characteristic, with two macrochaeta (Di1 and Di2) and a small microchaeta, sometimes indistinct (Di3). Abd. V with 2+2 or 3+3 tubercle, tubercle De is isolated from the tubercle Dl, or fused to tubercle Dl.

To date, 12 valid species are known in the genus *Lobellina* (Deharveng and Weiner 1984, Ma and Chen 2008). The new species can be distinguished from others by having six teeth on the mandible and fused tubercle Di on head. A key for all species of the genus is given below.

##### Key to species of genus *Lobellina*

**Table:** 

1	Cephalic chaeta O present	**2**
–	Cephalic chaeta O absent	**5**
2	Body macrochaetae smooth	**3**
–	Body macrochaetae serrate	**4**
3	Maxilla with 2 separate lamella and 3 teeth	***L. montana* Deharveng & Weiner, 1984 (Korea)**
–	Maxilla with 2 fused lamella and 2 teeth	***L. paraminuta* Deharveng & Weiner, 1984 (Korea)**
4	Body color yellow, mandible with 7 teeth, tubercle Oc with 2 chaetae, ventral tube with 5+5 chaetae, cephalic tubercle Di separate	***L. nanjingensis* Ma & Chen, 2008 (China)**
–	Body color red, mandible with 6 teeth, tubercle Oc with 3 chaetae, ventral tube with 4+4 chaetae, cephalic tubercle Di fused	***L. fusa* sp. n.**
5	Body macrochaetae smooth	**6**
–	Body macrochaetae serrate	**10**
6	Cephalic tubercle Oc with 3 chaetae	**7**
–	Cephalic tubercle Oc with 2 chaetae	**8**
7	Abd. V with 2+2 dorsal tubercles	***L. chosonica* Deharveng & Weiner, 1984 (Korea)**
–	Abd. V with 3+3 dorsal tubercles	***L. proxima* Deharveng & Weiner, 1984 (Korea)**
8	Tubercle Di on Abd. V with 2 chaetae	**9**
–	Tubercle Di on Abd. V with 3 chaetae	***L. minuta* (Lee, 1980) (Korea)**
9	Mandible with 3 teeth	***L. ipohensis* (Yosii, 1976) (Malaysia)**
–	Mandible with 8 teeth	***L. musangensis* (Yosii, 1976) (Malaysia)**
10	Cephalic tubercle Oc with 2 chaetae	**11**
–	Cephalic tubercle Oc with 3 chaetae	**12**
11	Abd. V with 2+2 dorsal tubercles	***L. ionescui* (Massoud & Gruia, 1974) (Cuba)**
–	Abd. V with 3+3 dorsal tubercles	***L. perfusionides* (Stach, 1965) (Vietnam)**
12	Abd. V with 3+3 dorsal tubercles	***L. kitazawai* (Yosii, 1969) (Japan)**
–	Abd. V with 2+2 dorsal tubercles	***L. roseola* (Yosii, 1954) (Japan)**

#### Genus *Paralobella* Cassagnau & Deharveng, 1984

##### Paralobella tianmuna sp. n.

Taxon classificationAnimaliaCollembolaNeanuridae

http://zoobank.org/CF024D84-DF1F-449E-ADBC-25E025FC585E

Figs 8–9 , 10–15 , Appendix 1 , Tables 2a –2b

###### Material.

Holotype, female, 3.0 mm. Chanyuan Temple, Tianmu Mountain, Zhejiang Province. Coordinates: 30°19'40"N, 119°26'15"E, ca. 390 m a.s.l., 30 July 2011, leg. Ji-Gang Jiang (Housed in Hunan University of Arts and Science, J2011073001). Paratypes: two males, three females and two juveniles, same data as holotype (Housed in Hunan University of Arts and Science, J2011073001); one female, Hengwu, Tianmu Mountain, Zhejiang Province. 30°19'41"N, 119°26'14"E, 400–500 m a.s.l., 27 July 2011, leg. Ji-Gang Jiang (Housed in Hunan University of Arts and Science, J2011072704).

###### Etymology.

The new species is named after the type locality, Tianmu Mountain.

###### Diagnosis.

Habitus typical of the genus *Paralobella*. Dorsal tubercles round or oval and well developed. 3+3 unpigmented eyes. Labrum round, chaetotaxy as 0/ 2, 2. Mandible with seven teeth. Maxilla nearly styliform, apex with two hook-like teeth. Cephalic tubercle Fr with chaeta O. Tubercle An with four chaetae, chaetae C and D free from the tubercle. Tubercle Oc with three chaetae. Cephalic tubercle Di, De, Dl respectively with 1, 3, 4 chaetae. Tubercle De of Th. II-III each with four (3+s) chaetae. Unguis with an inner tooth, and without lateral tooth. VT with 4+4 chaetae. Furcular vestige with three chaetae and no microchaetae.

###### Description.

Body length: male 2.0–2.5 mm; female 2.1–3.5 mm. Body entirely red while alive and white in alcohol.

**Figures 8–9.**
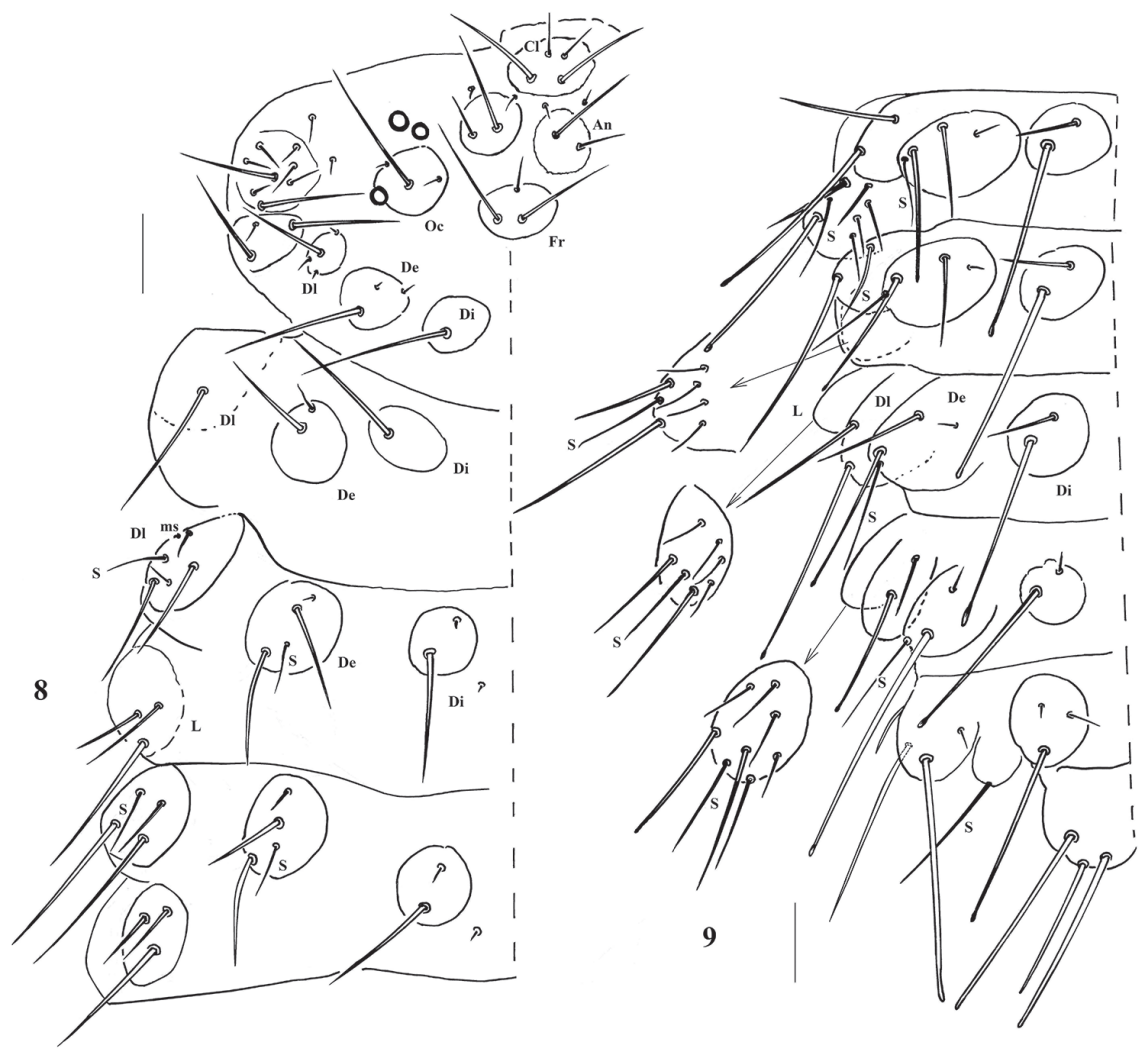
*Paralobella tianmuna* sp. n. **8** left half of head and thorax **9** left half of abdomen. Scale bar: 100 μm.

**Figures 10–15.**
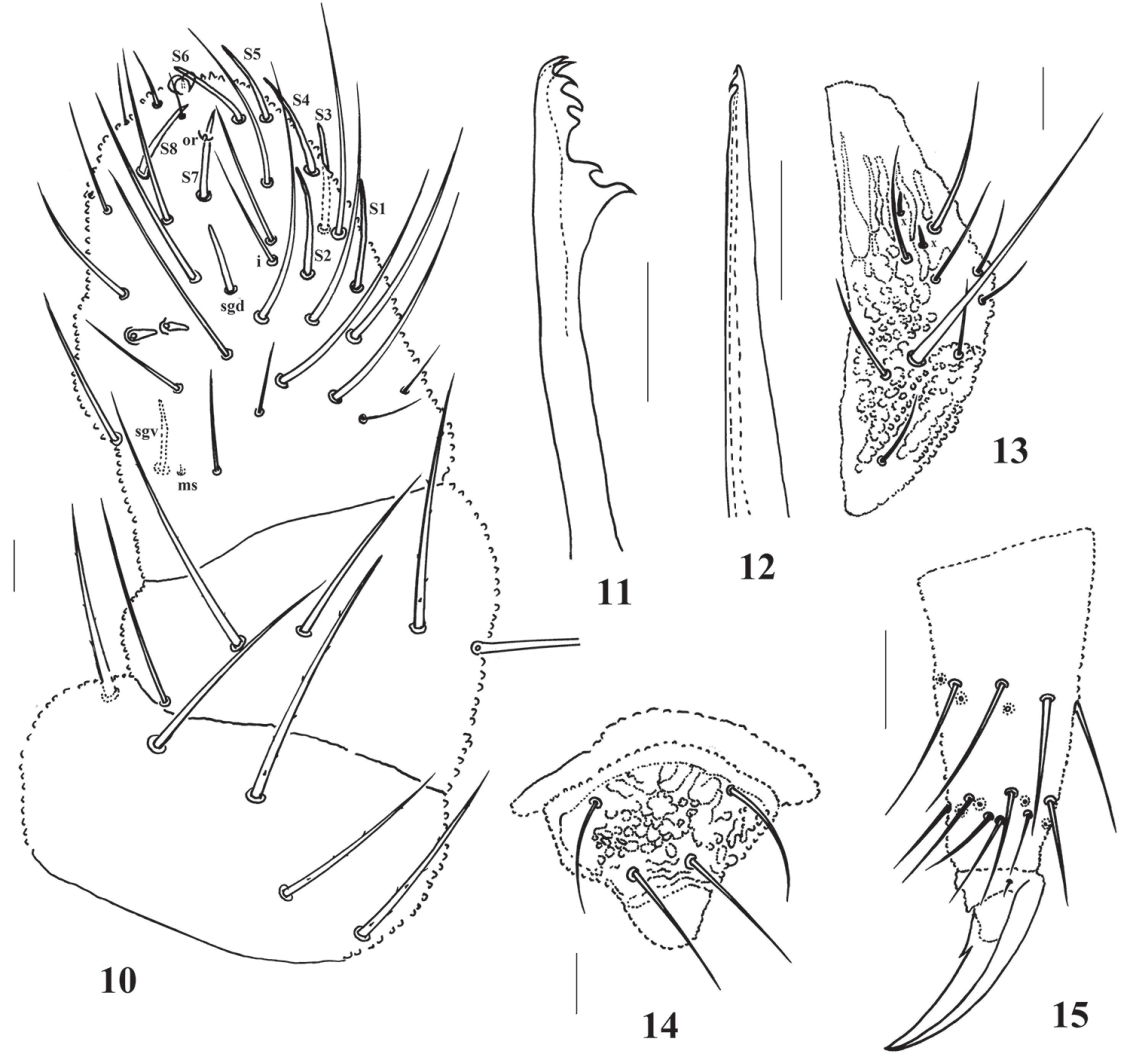
*Paralobella tianmuna* sp. n. **10** Antenna **11** Mandible **12** Maxilla **13** Labium **14** Labrum **15** Tibiotarsus and claw of hind leg. Scale bars: **10–14** 20 μm; **15** 50μm.

*Head*: Eyes 3+3 without pigment (Fig. 8). Ant. IV with trilobed apical bulb, and dorsal chaetotaxy as 8 sensilla, slender chaeta *i*, 12 sender, cylindrical chaetae and minute organite (Fig. 10). Labrum chaetotaxy as 0/ 2, 2 (Fig. 14). Labium with two minute distal x sensillum (Fig. 13). Mandible with seven teeth (Fig. 11). Maxilla nearly styliform, apex with two hook-like teeth (Fig. 12). Cephalic tubercles and chaetotaxy see Fig. 8 and Appendix 1, Table 2a. Macrochaetae on body weakly serrate, acuminate or with blunt apex. Mesochaetae and microchaetae smooth and pointed.

*Body tubercles* round or oval (Fig. 8–9). Chaetae Di3 free on the tubercles Di on Th. II-III. Abd. I-IV each with four tubercles. Abd. V with three tubercles, tubercle De only with an S-chaeta. The tubercles of Abd. VI with seven chaetae each. Body tubercles and chaetotaxy as in Appendix 1, table 2a and 2b.

*Appendages*: Unguis with an inner tooth, and without lateral tooth (fig. 15). VT with 4+4 chaetae. Furcular vestige with three chaetae and no microchaetae.

###### Remarks.

At present, 12 species belong to the genus *Paralobella*, all from Asia (Jiang et al. 2012, Luo and Palacios-Vargas 2016). In general appearance, *Paralobella tianmuna* sp. n. strongly resembles *P. perfusa* (Denis, 1934) from Indochina in the structure of maxilla, the number of mandible teeth, the arrangement of body tubercles, the presence of chaeta O of tubercle Fr, the tubercle Di of Th. I with one chaeta, and the tubercle De and Dl of Abd. V separate. However, *Paralobella tianmuna* sp. n. can be distinguished from the latter by number of chaetae on body, the former has 3 chaetae on each tubercle Di of Th. II–III, 7 (6+s) chaetae on each tubercle L of Abd. II-III; the latter has 2 chaetae on each tubercle Di of Th. II–III, 4 (3+s) chaetae on each tubercle L of Abd. II-III. The new species is also similar to Chinese species *P. breviseta* Luo & Palacios-Vargas, 2016 and *P. palustris* Jiang, Luan & Yin, 2012 in the arrangement of body tubercles, the presence of chaeta O of tubercle Fr, tubercle Di of Th. I with one chaeta, and the separate tubercle De and Dl of Abd. V. The new species can be separated from its congeners by the following key.

##### Key to species of genus *Paralobella* Cassagnau & Deharveng, 1984

**Table:** 

1	Chaeta O of tubercle Fr absent	**2**
–	Chaeta O of tubercle Fr present	**6**
2	Tubercle Dl of Th. II–III with 5 (4+s) chaetae (besides of ms)	***P. erawan* (Thailand)**
–	Tubercle Dl of Th. II–III with 4 (3+s) chaetae (besides of ms)	**3**
3	Tubercle Di of Th. II–III only with 1 chaeta, tubercle Oc on head only with Ocm chaeta	***P. selangorica* (Malaysia)**
–	Tubercle Di of Th. II–III with 2–3 chaetae, tubercle Oc on head with 2–3 chaetae	**4**
4	Tubercle Di of Th. II–III with 2 chaetae	***P. penangensis* (Malaysia)**
–	Tubercle Di of Th. II–III with 3 chaetae	**5**
5	Abd. I–III: tubercle De with 5 (4+s) chaetae, tubercle Dl with 3 chaetae; mandible complicated, totally with 16 teeth in two rows	***P. apsala* (Thailand)**
–	Abd. I–III: tubercle De with 4 (3+s) chaetae, tubercle Dl with 2 chaetae; mandible relatively simple, with 5 or 6 teeth in one row	***P. kinabaluensis* (Malaysia)**
6	Tubercle De of Th. II–III with 4 (s+3) chaetae, VT with 3+3 chaetae	***P. sabahna* (Malaysia)**
–	Tubercle De of Th. II–III with 5 (s+4) chaetae, VT with 4(5)+4(5) chaetae	**7**
7	Tubercle Di, De, DL of Th. I respectively with 2, 2, 1 chaetae	**8**
–	Tubercle Di, De, DL of Th. I respectively with 1, 2, 1 chaetae	**9**
8	Tubercle Dl of Th. II–III with 4 (3+s) chaetae, tubercle Dl of Abd. IV with 6 (5+s) chaetae	***P. khaochongensis* (Thailand)**
–	Tubercle Dl of Th. II–III with 5 (4+s) chaetae, tubercle Dl of Abd. IV with 3 (2+s) chaetae	***P. paraperfusa* (Philippines)**
9	Tubercle De and Dl of Abd. V fused, body tricolour	***P. orousseti* (Philippines)**
–	Tubercle De and Dl of Abd. V separate, body red color	**10**
10	Mandible with 20 teeth	***P. palustris* (China)**
–	Mandible with less than 8 teeth	**11**
11	Mandible with 6 teeth, tubercle L of Abd. II-III with 6 (5+s) chaetae	***P. breviseta* (China)**
–	Mandible with 7 teeth, tubercle L of Abd. II-III with 4 (3+s) or 7 (6+s) chaetae	**12**
12	Tubercle Di of Th. II–III with 2 chaetae, tubercle L of Abd. II-III with 4 (3+s) chaetae, mandibular basal tooth much larger than the second tooth	***P. perfusa* (Indochina)**
–	Tubercle Di of Th. II–III with 3 chaetae, tubercle L of Abd. II-III with 7 (6+s) chaetae, mandibular basal tooth slightly larger than the second one	***P. tianmuna* sp. n.**

## Supplementary Material

XML Treatment for Crossodonthina bidentata

XML Treatment for Lobellina fusa

XML Treatment for Paralobella tianmuna

## Appendix 1

**Table 1a.** Cephalic tubercles and chaetotaxy of *Lobellina fusa* sp. n.

Tubercle	Number and type of chaeta	Names of chaeta
Cl	2 Ml	F
	2 Mcc	G
An	2 Ml2 mi	B, EC, D
Fr	2 Ml1 mc	AO
Oc	2 Ml1 mi	Oca, OcmOcp
Di+Di	1+1 Ml	Di1
De	1 Ml+1 Mc1 mi	De1, De2De3
Dl	2 Ml3 mi	Dl1, Dl5Dl2, Dl3, Dl6
L	1 Ml1 Mc	L1Dl4
So	2 Ml+6 me5 me	uncertain

**Table 1b.** Body tubercles and chaetotaxy of *Lobellina fusa* sp. n.

**Terga**					**Legs**				
	Di	De	Dl	L	Scx2	Cx	Tr	Fe	T
Th.I	1	2	1	–	0	3	6	12	19
Th.II	3	4+s	3+s+ms	3	2	6	7	9	19
Th.III	3	4+s	3+s	3	2	8	6	10	18
**Terga**					**sterna**				
Abd. I	2	3+ s	2	4	VT	4			
Abd. II	2	3+ s	2	5	Ve	2(4)	Vl	–	
Abd. III	2	3+ s	2	5	Ve	3	Fu	3	
Abd. IV	2	2+ s	3	7	Ve	7	Vl	5	
Abd. V	3	s	4		Ag	3	Vl	1	
Abd. VI	7				Ve	14	Ve	5	

**Table 2a.** Cephalic tubercles and chaetotaxy of *Paralobella tianmuna* sp. n.

Tubercle	Number and type of chaeta	Names of chaeta
Cl	2 Mc	F
	2 Mcc	G
An	1 Mc	B
	1 Mcc	E
	2 me	C, D
Fr	2 Mc	A
	1 Mcc	O
Oc	1Ml	Ocm
	2 me	Oca, Ocp
Di	1 Ml	Di1
De	1 Ml	De1
	2 me	De2, De3
Dl	1 Mc	Dl1,
	3 me	Dl3,Dl5, Dl6
L	2 Mc	L1, L4
	1 me	L3
So	2 Mc	uncertain
	8 me	

**Table 2b.** Body tubercles and chaetotaxy of *Paralobella tianmuna* sp. n.

Terga					Legs					
	Di	De	Dl	L	Scx2	Cx	Tr		Fe	T
Th.I	1	2	1	–	0	3	6		13	19
Th.II	3	4+s	4+s+ms	3	2	7	6		12	19
Th.III	3	4+s	4+s	3	2	8	6		11	18
**Terga**					**sterna**					
Abd. I	2	3+ s	2	6+s	VT	4				
Abd. II	2	3+ s	2	6+s	Ve	4		Vl	–	
Abd. III	2	3+ s	2	6+s	Ve	4		Fu	3	
Abd. IV	2	2+ s	3	7+s	Ve	7(8)		Vl	5	
Abd. V	3	s	4		Ag	3		Vl	2	
Abd. VI	7				Ve	14		Ve	6	
